# Differing patterns of stress and craving across the day in moderate-heavy alcohol consumers during their typical drinking routine and an imposed period of alcohol abstinence

**DOI:** 10.1371/journal.pone.0195063

**Published:** 2018-04-18

**Authors:** Rhiannon E. Mayhugh, W. Jack Rejeski, Meredith R. Petrie, Paul J. Laurienti, Lise Gauvin

**Affiliations:** 1 Laboratory for Complex Brain Networks, Wake Forest School of Medicine, Winston-Salem, North Carolina, United States of America; 2 Neuroscience Program, Wake Forest School of Medicine, Winston-Salem, North Carolina, United States of America; 3 Department of Health and Exercise Science, Wake Forest University, Winston-Salem, North Carolina, United States of America; 4 Department of Psychology, Wake Forest University, Winston-Salem, North Carolina, United States of America; 5 Translational Science Center, Wake Forest University, Winston-Salem, North Carolina, United States of America; 6 Department of Geriatric Medicine, Wake Forest University, Winston-Salem, North Carolina, United States of America; 7 Department of Radiology, Wake Forest School of Medicine, Winston-Salem, North Carolina, United States of America; 8 Centre de recherche du Centre Hospitalier de l’Université de Montréal (CRCHUM) & Département de médecine sociale et préventive, École de Santé Publique, Université de Montréal, Montréal, QC, Canada; Harvard Medical School, UNITED STATES

## Abstract

**Background:**

Stress is a known factor related to alcohol use. However, how the relationship between alcohol craving and stress varies across the day is not fully understood. As craving is a consistent predictor of alcohol use disorder (AUD), understanding stress and craving patterns across the day in routine, non-dependent, moderate-heavy alcohol consumers may help in understanding those who may be vulnerable to transitioning into AUD.

**Method:**

Moderate-heavy drinkers were recruited from the local community (n = 32) and assessed for fluctuations in craving and stress intensity across the day via Ecological Momentary Assessment (EMA) during 3 consecutive days of imposed alcohol abstinence (abstained trial) and their normal drinking routine (normal trial). A multilevel modeling statistical approach assessed differences in diurnal craving and stress patterns with the Alcohol Craving Experience Questionnaire (ACE) examined as a moderator.

**Results:**

Immediately following alcohol consumption on normal trials, EMA craving levels were significantly reduced compared to pre-drinking levels. Moreover, the post-drinking craving levels were lower than on abstained trials. Higher ACE scores were associated with significantly higher EMA craving across the day and higher peaks at midday. Higher ACE scores were also associated with greater EMA stress across the day. Drinking relieved stress relative to abstained trials, but not in individuals with higher ACE scores. Higher stress was associated with greater EMA craving, which was stronger among those with higher ACE scores.

**Conclusion:**

These findings suggest that ACE scores are important to understanding patterns of stress and craving experienced across the day in routine, non-dependent, moderate-heavy drinkers and may provide new insights for vulnerability to transitioning into AUD.

## Introduction

The impact of stress on public health has become a major concern. A 2015 national survey showed that one-third of adults reported higher stress levels over the previous year with a significantly higher proportion reporting extreme stress [1]. One major concern related to rising stress levels is the elevated risk for Alcohol Use Disorder (AUD) and relapse in those suffering from addiction [2]. Although stress is a known factor in motivation to drink, the alcohol-stress relationship is complex, influenced by diverse environmental and biological factors, and changes along the transition to addiction [3, 4].

Craving for alcohol has been associated with consumption and is a subjective marker of AUD [5, 6]. For example, craving intensity across the day predicted greater number of drinks consumed in both dependent and non-dependent heavy alcohol drinking adults [7, 8]. The Elaborated Intrusion (EI) Theory of Desire views craving as a cognitive-emotional event consisting of sensory imagery episodes that vary in duration and intensity [9]. EI theory emphasizes negative affect, a common correlate of stress [10], as central to triggering intrusive desires for substances such as alcohol. Negative affect also increases awareness of any physiological deficit or feeling of deprivation, motivating people to approach activities, such as alcohol consumption, as a means of rebalancing one’s hedonic state [9]. Indeed Cooney et al. have observed that, in men with alcoholism undergoing inpatient treatment, greater urge to drink when exposed to negative mood imagery and alcohol cues predicted shorter time to relapse after discharge [11].

The current study investigated the stress-craving relationship throughout the day in routine moderate-heavy alcohol consumers during periods of both drinking in their normal routine (normal trial) and an imposed period of alcohol abstinence (abstained trial). We also examined the acute effects of alcohol consumption by evaluating before drinking (pre-drinking) compared to after consuming alcohol (post-drinking) on normal trial days. Triggers of stress and craving are dependent on contextual, temporal, and environmental factors that vary across the day [12–14]. To capture these variations in stress and craving across the day this study utilized Ecological Momentary Assessment (EMA) methodology. This method involves repeated measurements, typically utilizing portable technology, of a subject’s experience occurring in real time within their natural environment.

To understand how craving and stress across the day relates to an individual’s trait craving characteristics, participants were also assessed with the Alcohol Craving Experience Questionnaire (ACE). The ACE is based on EI Theory of Desire and is an established measure of craving known to effectively discriminate between high and low risk drinkers and clinical versus non-clinical populations [15]. This study chose the ACE for this discriminative power and, although we did not expect extremely high scores within this group, it allowed us to classify individuals into lower and higher trait craving.

ACE scores were assessed for associations with EMA stress and craving patterns across the day. Based on EI Theory of Desire’s view that the sensory-imagery experience behind craving is cumulative with additional cue exposure, we hypothesized that craving would increase across the day on both normal and abstained trials (hypothesis 1). We also expected higher alcohol craving across the day on the abstained trial, as drinking was not an available option to relieve the unfulfilled craving for alcohol (hypothesis 2a). In a more fine-grained analysis of abstinence and craving, we stratified responses on the normal trial as a function of whether they occurred prior to or following a drink. We expected that EMA craving would be substantially higher pre-drinking than post-drinking (hypothesis 2b) and that ratings would be greater across the day among those scoring higher on the ACE (hypothesis 3). Most importantly, due to the impact of negative affect on craving, we expected participants with higher ACE scores to report higher stress across the day (hypothesis 4a) and to exhibit a stronger relationship between EMA stress and craving (hypothesis 4b). Given limited previous research on EMA stress as a function of ACE scores, we did not formulate hypotheses regarding the association of time of day and drinking on stress and as a function of ACE scores.

In summary, the overarching goal of the current study was to investigate the experience of stress and craving across the day in moderate-heavy alcohol consumers during routine drinking behavior and imposed abstinence, and to determine if the relationship between these measures differed as a function of ACE scores. These findings are important, not only in understanding the experience of stress and craving across the day in routine, non-dependent drinkers, but in assessing the impact of drinking (acquisition of the target of craving), or lack thereof (abstinence) on stress and craving; a key manipulation from the perspective of the EI Theory of Desire.

## Materials and methods

### Participants

We recruited thirty-four participants from the greater Winston-Salem, North Carolina area as part of a larger study that included brain imaging, which was approved by the Wake Forest University Health Sciences IRB. We excluded two participants due to missing EMA data, resulting in a final sample of 32 participants (14 men and 18 women). Inclusion criteria required that participants (a) were 24–60 years old, (b) consumed alcohol ≥ 50% of days in the past 3 months, and (c) maintained an average daily alcohol consumption of 1–3 drinks/day for women and 2–4 drinks/day for men for ≥ the past three years. Exclusion criteria included (a) previous or current diagnosis of AUD; (b) binge drinking as defined by the National Institute on Alcohol Abuse and Alcoholism (≥ 4 drinks for females, ≥ 5 drinks for males within 2 hours [16] more than once a month; (c) ˃ 3 occurrence of consuming alcohol before noon in the past 3 months; (d) currently undergoing treatment for a serious illness (diagnosed depression allowed if treated and stable for at least 2 months); (e) scored ˃ 20 on the Center for Epidemiological Studies Depression Scale (CES-D); (f) a neurological disease diagnosis; (g) consuming ≥ 500 mg/day of caffeine; (h) smoking more than 1.5 packs/day; or (i) a positive urine drug screen (Methamphetamine, Cocaine, Marijuana, Amphetamine, Opiates, & Benzodiazepines). Due to the association between body mass index (BMI) and blood-alcohol content (BAC), BMI was restricted to 18.5 kg/m^2^ to ≤ 35 kg/m^2^ [17]. Finally, because this study was part of a larger MRI project, participants had to be right-handed, not claustrophobic, or have any other medical condition that would put them at risk during the scanning protocol. The Clinical Institute Withdrawal Assessment of Alcohol (CIWA-Ar) was used as a screening measure for alcohol withdrawal symptoms on the abstained trial [18], which also served as a check for physical dependence (positive result never occurred). Participants received a total of $350 for study completion.

### Procedure

#### Study overview

**Fig 1:**
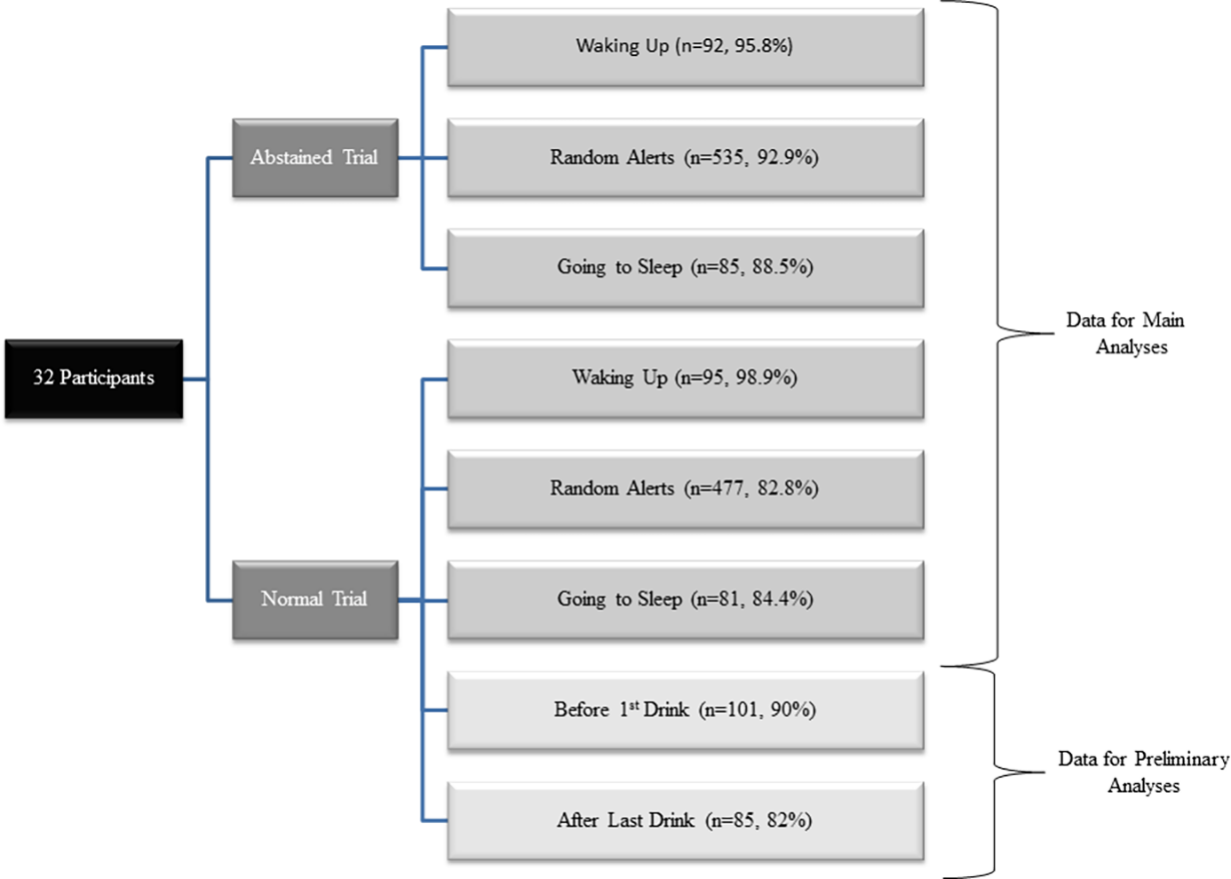
Design of the study, stimuli for Ecological Momentary Assessment (EMA) recordings, and compliance.

The study protocol consisted of an initial screening visit and two experimental trials: three consecutive days of a normal drinking routine (normal trial), and three consecutive days of abstaining from alcohol (abstained trial). We scheduled both trials (normal and abstained) to ensure participants had no major disruptions (i.e. medical procedures) to their typical drinking routines or events that might impede the three consecutive days of alcohol abstinence, while randomizing participants to the order in which they received the normal and abstained trials. All study trials were scheduled to avoid any major life stressors or atypical life events. EMA data were collected throughout the three days of each experimental trial (Fig 1). Although not the focus of this report, both three consecutive trial days were followed by an MRI scan appointment (4^th^ day). A urine drug test (see the Participants section above for details) was administered at the beginning of each MRI appointment which ensured the absence of drug use during the extent of study participation.

#### Study measures

We employed the Time Line Follow Back (TLFB) [19], modified to record time of day (morning, afternoon, and evening) of consumption, to quantify alcohol consumption over the previous three months. This measure was used to verify that individual’s drinking patterns met the eligibility criteria and later to describe the sample’s drinking behavior (Table 1). The EMA protocol utilized a Likert measure to assess intensity of craving and stress asking: “Do you have a craving for alcohol right now?” or “How much stress do you feel right now?” The response scale ranged from 0–10, with 0 for “no craving/no stress” and 10 for “extreme craving/extreme stress.” In addition, participants self-administered a breath test for alcohol content after each Likert survey (see EMA protocol section below). This increased confidence that participants maintained alcohol abstinence during the abstained trial. We administered the ACE questionnaire following both the normal and abstained trials to capture participants’ perceived intensity of craving across the past 3-days of each trial. In addition, we used the ACE strength of craving questionnaire following both trials using a “right now” or state-based response set because our interest was to use data from the strength measure in conjunction with functional magnetic resonance imaging (fMRI) data collected following each 3-day trial. The analyses presented here are restricted to the 11-item ACE frequency measure as a potential moderator of EMA responses collected during the two 3-day trials. Published psychometric data on the ACE supports its validity and reliability [15, 20]. In the current study, we also computed an alpha internal consistency reliability on the scale during both 3-day trials. An internal consistency reliability of 0.94 for the normal trials and 0.89 for the abstained trials support our using a composite total score for the ACE frequency questionnaire. Because there was no statistically significant difference between scores on the ACE frequency questionnaire assessed following either the normal or abstained trials (p = 0.34), we averaged values across the two trials for use in our analyses.

**Table 1:** 

Variable	Overall (n = 32)	Male (n = 14)	Female (n = 18)
Age	38.28 (10.8)	37.29 (6.8)	39.06 (13.2)
Body Mass Index	25.02 (3.9)	26.03 (3.7)	24.23 (4.0)
Race			
Black or African American	3	2	1
Asian	1	1	0
White	28	11	17
Alcohol Use			
Total Years Drinking	18.22 (10.8)	17.86 (7.2)	18.50 (13.2)
Years Maintained Current Drinking Pattern	8.06 (5.9)	9.29 (5.4)	7.11 (6.2)
Previous 3 Months (Time Line Follow Back)			
% of days that were drinking days	80.79 (15.6)	79.53 (16.1)	81.77 (15.6)
Average drinks consumed on drinking days	2.26 (0.7)	2.37 (0.3)	2.17 (0.9)

Sample Characteristics. Males and females did not differ significantly in any of the above categories (alpha = 0.05).

#### Participant screening

A phone screen served as the initial evaluation of eligibility, followed by an in-person screening visit that included informed consent, a final determination of eligibility, and completion of self-report questionnaires including the ACE Questionnaire [20].

#### The EMA protocol

EMA data collection occurred with study-issued iPhones that had three smartphone applications. iSurvey by Harvest Your Data (Wellington, New Zealand) allowed participants to complete a single item EMA craving and stress assessment. Audible Alerts (Wake Forest Medical Center Health Sciences) dispatched these random alerts to study participants prompting an EMA assessment. Breathometer brand (www.breathometer.com, Burlingame, CA), then BACtrack Mobile Pro (for improved functionality, www.bactrack.com, San Francisco, CA,) testing devices helped ensure alcohol abstinence.

Participants completed EMA assessments upon waking each morning, going to bed each evening, and when randomly prompted (9am-9pm) during both the normal and abstained trials. During normal trials, assessments occurred immediately before the first drink and after the last drink. The waking, going to bed, prior to the initial drink, and following the final drink of a drinking episode on normal trial assessments contributed to data points outside the 9am-9pm window. For each EMA assessment, participants first recorded the reason for the EMA assessment (“I just woke up”, “I received a random alert”, “I am about to start drinking”, “I have just finished drinking”, or “I am going to sleep”). They then completed the assessment by sliding a bar to the appropriate location on the Likert scale to indicate their current level of craving and stress, followed by a BAC breath test.

#### Statistical analysis strategy

EMA reports from all participants were stacked and the number of responses was tallied as a function of whether they occurred on abstained or normal trials. EMA recordings during the normal trials were further stratified as occurring either prior to (pre-drinking) or following (post-drinking) a drink. Time of day was transformed into military time and centered at 15:00 hours (3 PM). EMA reports recorded after midnight, but before retiring for the night, were given values above 24:00 hours to accurately reflect the day on which they occurred (e.g., 25:00 for 1 AM). To account for nonlinear variations in levels of craving associated with time of day, the centered value of military time was squared to operationalize quadratic trends. Within participant variables were created to distinguish the abstained trials from the normal trials (i.e., the main study manipulation). One set of within participant variables was used to contrast EMA responses occurring on abstained vs. normal trials. Another set of within participant variables was used for a more fine-grained analysis of the effects of alcohol consumption on normal trials. The first contrasted occasions on normal trials following the occurrence of the first drink (post-drinking, value of 1) to all other occasions (value of 0). The second contrasted occasions on normal trials after the occurrence of the first drink (pre-drinking occasions, value of 1) to all other occasions. For between participant variables, a categorical variable was used for sex (women = 1, men = 0) and ACE scores were centered at the sample mean. Descriptive analyses were performed with SPSS (version 19).

The distribution of craving reports was examined for normality and was found to exhibit positive skewness. Attempts to normalize the distribution by performing square root and logarithmic transformations were unsuccessful. To ensure that the skewness did not meaningfully impact the results, additional analyses were conducted using different operational definitions of the craving variable that were less skewed (for example, modeling the data with a Poisson distribution). The results of these analyses were identical for pre-drinking and post-drinking vs. abstained trials, the effects of time of day, and abstained trials vs. normal trials. The effects of ACE and female sex did however vary somewhat across these analyses possibly due to the relatively small sample size. Given that the main findings were not sensitive to the operational definitions of the craving variable, the findings presented in the current manuscript were limited to the main analysis with the craving score treated as a continuous variable.

Given the nested structure of the data set (i.e., repeated measures of cravings on abstained and normal trials which were nested within respondents) and the interest in ascertaining the influence of both within and between participant variables, the main statistical analysis strategy consisted of growth curve analyses, also known as multilevel modeling (Raudenbush, 2001). Multilevel modeling was performed with HLM (version 7.0, Scientific Software International, Chicago, IL). In a first set of analyses as a manipulation check, EMA responses occurring immediately before the first drink and immediately after the participant indicated that they had finished drinking (last drink) were extracted from the data set. A null multilevel regression model was applied to the extracted data set to estimate the intraclass correlation coefficient (ICC) (proportion of the total variance attributable to between person variation) and to test whether or not the between participant variance in overall craving was statistically significant. Then, the variable contrasting EMA reports collected immediately before the 1^st^ drink and after the last drink investigated the effect of consuming alcohol on craving.

All subsequent analyses were performed on the dataset from which recordings immediately prior to the first drink and following the last drink were removed from the normal trial (not necessary in the abstained trial). A null model was applied to estimate the ICC and to test the statistical significance of the between participant variance. Then, the variables operationalizing the linear and quadratic trends of time throughout the day were entered into the model to explore within day variations in craving intensity. Then, the variable contrasting EMA reports collected on abstained trials in comparison to normal trials was entered into the model to examine the effects of the study’s main experimental manipulation.

A separate set of analyses, which was similar to the previous, was then conducted using two variables to operationalize the effects of drinking on normal trials. That is, the two variables operationalizing effects of pre-drinking and post-drinking on normal trials in comparison to abstained trials were entered into the model. This allowed for a more fine-grained examination of craving as a function of the study’s experimental manipulation alongside the effects of alcohol consumption. Finally, the between participant variable operationalizing sex and ACE scores were added as a moderator of overall craving, time of day, abstaining, and alcohol consumption. Results were plotted to better illustrate findings.

Next, we investigated stress as an outcome variable in order to examine the effects of time of day, abstaining from drinking, and consumption of alcohol. We performed multilevel modeling by entering the effects of time of day (linear and quadratic trends), as well as the variables operationalizing alcohol consumption, in comparison to abstained trials and the moderating effects of ACE scores. Again, results were also plotted to illustrate findings. In a final set of models, we entered the effects of time of day, alcohol consumption, and stress while adjusting for the moderating influence of ACE on each of these variables.

## Results

The 34 participants provided a total of 1602 responses to the EMA craving assessment. Data from two participants were removed because one participant did not complete the ACE measure (n = 47 craving responses removed) and the other did not comply with the EMA procedure (n = 4 craving responses removed). The final data set included 1551 EMA responses recorded from 32 included participants. Among the 1551 EMA reports, 1365 were elicited either because the person had just awakened in the morning (n = 187), because s/he was about to go to bed (n = 166), or because s/he received an alert to complete an assessment (n = 1012). Of the 1365 reports, 712 occurred on abstained trials and 653 on normal trials (508 pre-drinking and 145 post-drinking). Another 186 EMA reports were triggered by the decision to drink, with 101 reports occurring immediately before the first drink and 85 after the last drink. Given that some participants experienced more than one drinking episode on normal trials, they provided more of these ancillary reports than requested. Individual participants provided between 34 and 48 EMA responses in response to getting up, going to bed, or random alerts (M = 42.7, SD = 3.5) and between 1 and 13 responses prior to and following drinks and during normal trials (M = 5.8, SD = 2.3).

Compliance with the EMA procedure for each of the different response conditions appears in Fig 1. As can be seen, compliance was high ranging from 82% (random alerts on normal trials) to 98.9% (waking up on normal trials). Abstinence from alcohol consumption on the abstained trials was confirmed both verbally and by random breath testing for all participants. On the three occasions when the breath test device malfunctioned, participants contacted the study coordinator and device troubleshooting was conducted. To encourage honesty, the participant could reschedule the abstained trial days without penalty in the event that a drink was consumed (this was never necessary). Although alcohol abstinence could not be confirmed with 100% certainty, we believe that the breath test protocol and communication with the study coordinator served to greatly enhance the confidence that the abstained state was maintained. For the entire sample, there were only three days on which participants reported consuming no alcohol during the normal trial. These occasions occurred in three separate individuals (all participants drank at least 2/3 of the drinking days) increasing confidence that the 3-day abstinence period disrupted normal drinking routines. Although the recruitment criteria required that alcohol be consumed on at least 50% of days on the TLFB, the actual sample reported drinking on 80.8% of days. Table 1 provides participant characteristics including age, BMI, race, and alcohol use. It should be noted that the sample included slightly more women (56.6%) and averaged ACE scores ranged from 0 to 4.23 and varied substantially (M = 1.14, SD = 1.02, 95% CI [0.73, 1.50]).

In the first set of analyses, the null model applied to the EMA data set immediately before the 1^st^ drink and immediately after the last drink (n = 186 reports nested within 32 participants) revealed statistically significant between participant variance in the craving intensity (X^2^(31) = 130.72, p<0.001). The ICC was estimated to be p = 0.33 indicating that 67% of the variance in craving intensity was within person variation whereas 33% was between participant variation. The intensity of cravings were significantly higher (p<0.001) pre-drinking in comparison to post-drinking (predicted values = 4.45 vs. 1.06) supporting the well-known effects of alcohol in reducing craving.

The null model applied to the data set involving the remaining 1365 EMA reports also showed statistically significant between participant variance craving (X^2^(31) = 809.9, p<0.001). The ICC was estimated to be p = 0.37 again suggesting that most of the craving was within person variation. Modeling of the effects of time showed that craving increased as the day progressed but then dropped off later in the day (linear effects: b = 0.14, SE = 0.01, p <0.001; quadratic effects b = -0.022, SE = 0.002, p <0.001), supporting hypothesis 1. Further, modeling showed that craving was significantly higher on abstained trials in comparison to normal trials (b = 0.20, SE = 0.10, p< 0.045), also in support of hypothesis 2a.

**Fig 2:**
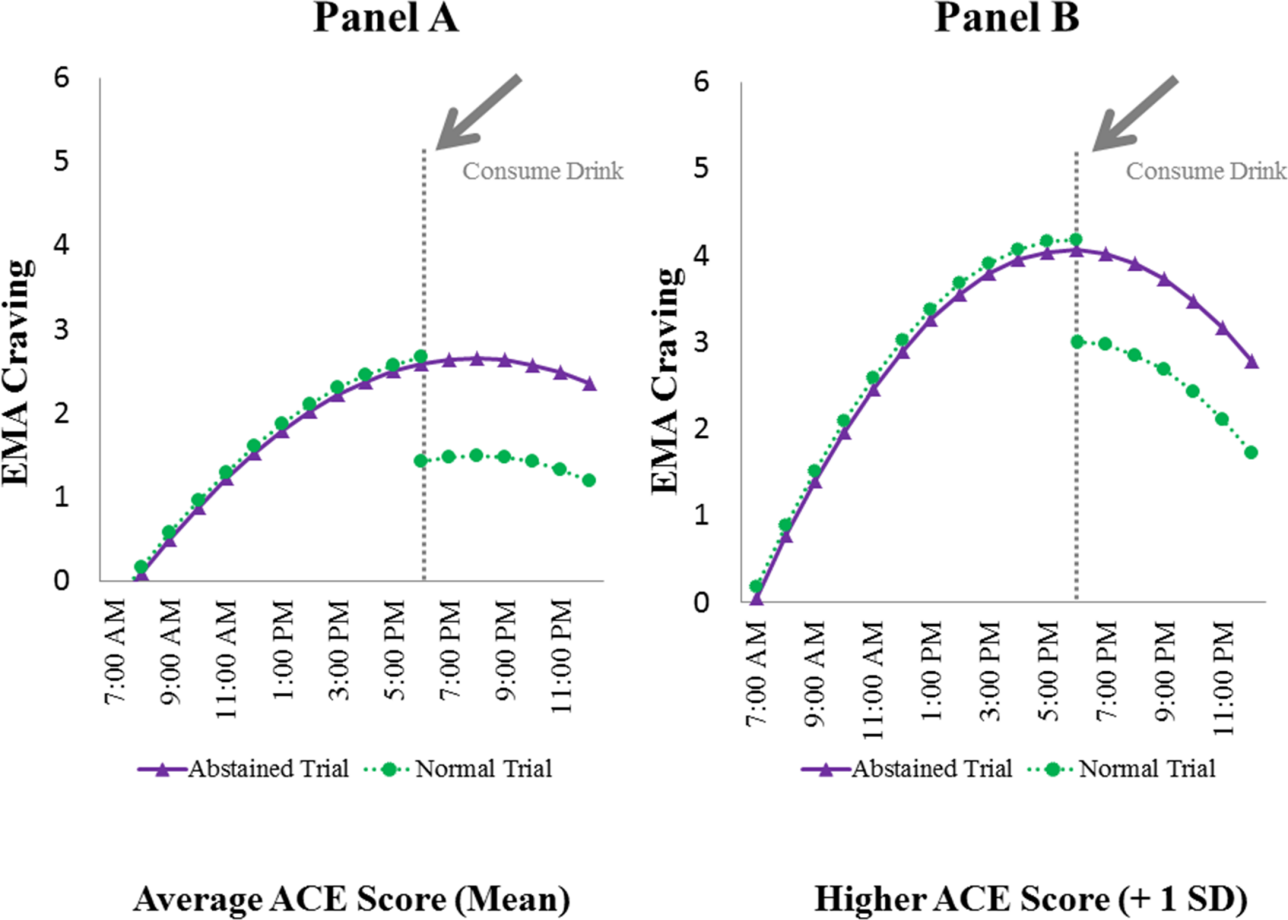
EMA craving in average and higher ACE scores. Predicted craving scores as a function of time of day, normal or abstained trials, and alcohol consumption across individuals with average (Panel A) and higher ACE scores (Panel B).

To further elucidate these findings, the next set of analyses jointly examined the influence that the experimental manipulation (alcohol abstinence) and alcohol consumption had on cravings. Once the effects of the time of day were included in the model, we observed that pre-drinking EMA reports of craving (on normal trials) were not different (p>0.05) from EMA reports recorded at comparable times on abstained trials. However, post-drinking EMA craving reports were significantly lower (b = -1.08, SE = 0.19, p<0.001) than both pre-drinking and abstained trial EMA reports, supporting hypothesis 2b. These findings are illustrated in Fig 2, Panel A. For hypothesis 3, we observed that individuals with higher ACE scores showed unique patterns of diurnal variations in craving throughout the day (Fig 2, Panel B). That is, these individuals reported higher EMA craving throughout the day (b = 1.57, SE = 0.18, p<0.001) and reached higher levels at midday (b = 0.02, SE = 0.002, p<0.001). ACE scores had no effect on craving reported on abstained versus normal trials; also, regardless of ACE scores, participants reported similar decreases in craving as a result of drinking (see Fig 2, Panels A & B).

**Fig 3:**
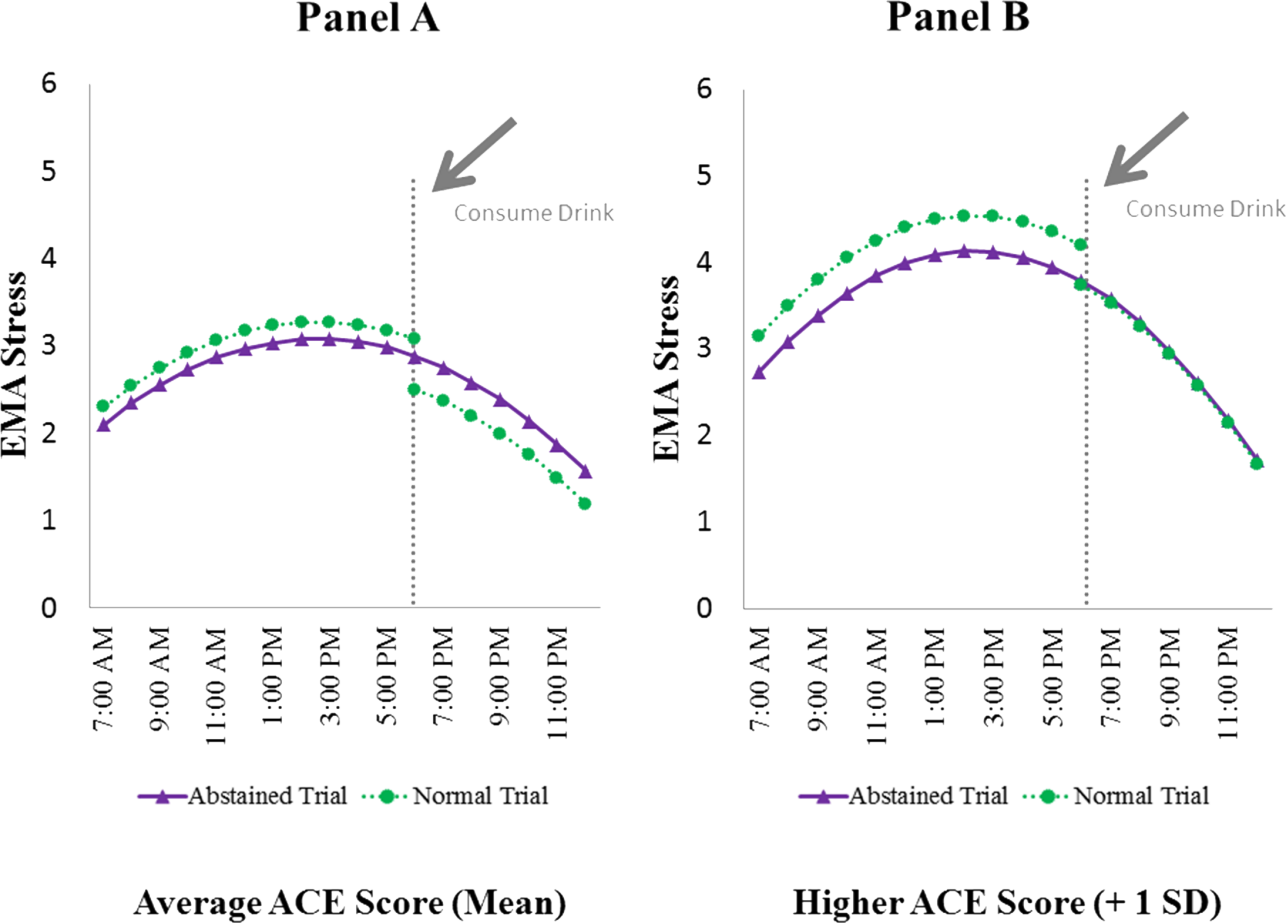
EMA stress in average and higher ACE scores. Predicted stress scores as a function of time of day, normal or abstained trials, and alcohol consumption across individuals with average (Panel A) and higher ACE scores (Panel B).

Examination of within-day patterns of stress and the effects of alcohol consumption while adjusting for the moderating influence of ACE scores revealed unique findings (see Fig 3). The ICC for stress was substantially higher than for craving with p = 0.50 indicating that about 50% of variability in stress was between participant variability with the remaining variance being within person. For craving, the ICC was lower at about p = 0.37. Stress showed within-day variations that were different from those observed for craving. That is, stress levels were lowest early and late in the day and thus showed an inverted-U pattern throughout the day (linear trend: p>0.05; quadratic trend: b = -0.017, SE = 0.002, p<0.001). More interesting, participants’ ACE scores moderated the pattern of variation in stress throughout the day and as a function of alcohol consumption. That is, as illustrated in Fig 3, participants with higher ACE scores reported significantly greater stress throughout the day (moderating effect of ACE on the intercept: b = 1.04, SE = 0.26, p<0.001) along with a more accentuated inverted-U pattern (hypothesis 4a) (moderating effects of ACE on quadratic trends: b = -0.009, SE = 0.001, p<0.001). In addition, those with higher ACE scores reported elevated stress levels pre-drinking in comparison to comparable times on abstained trials (moderating effect of ACE: 0.22, SE = 0.10, p<0.04). Interestingly though, individuals with average ACE scores experienced a decrease in stress post-drinking compared to abstained trials (b = -0.38, SE = 0.17, p<0.024; see Fig 3A) but the moderating influence of ACE nullified this effect (b = 0.34, SE = 0.14, p<0.018)) such that individuals with higher ACE scores did not show this significant decrease post-drinking in comparison to abstained trials (see Fig 3, Panel B).

**Fig 4:**
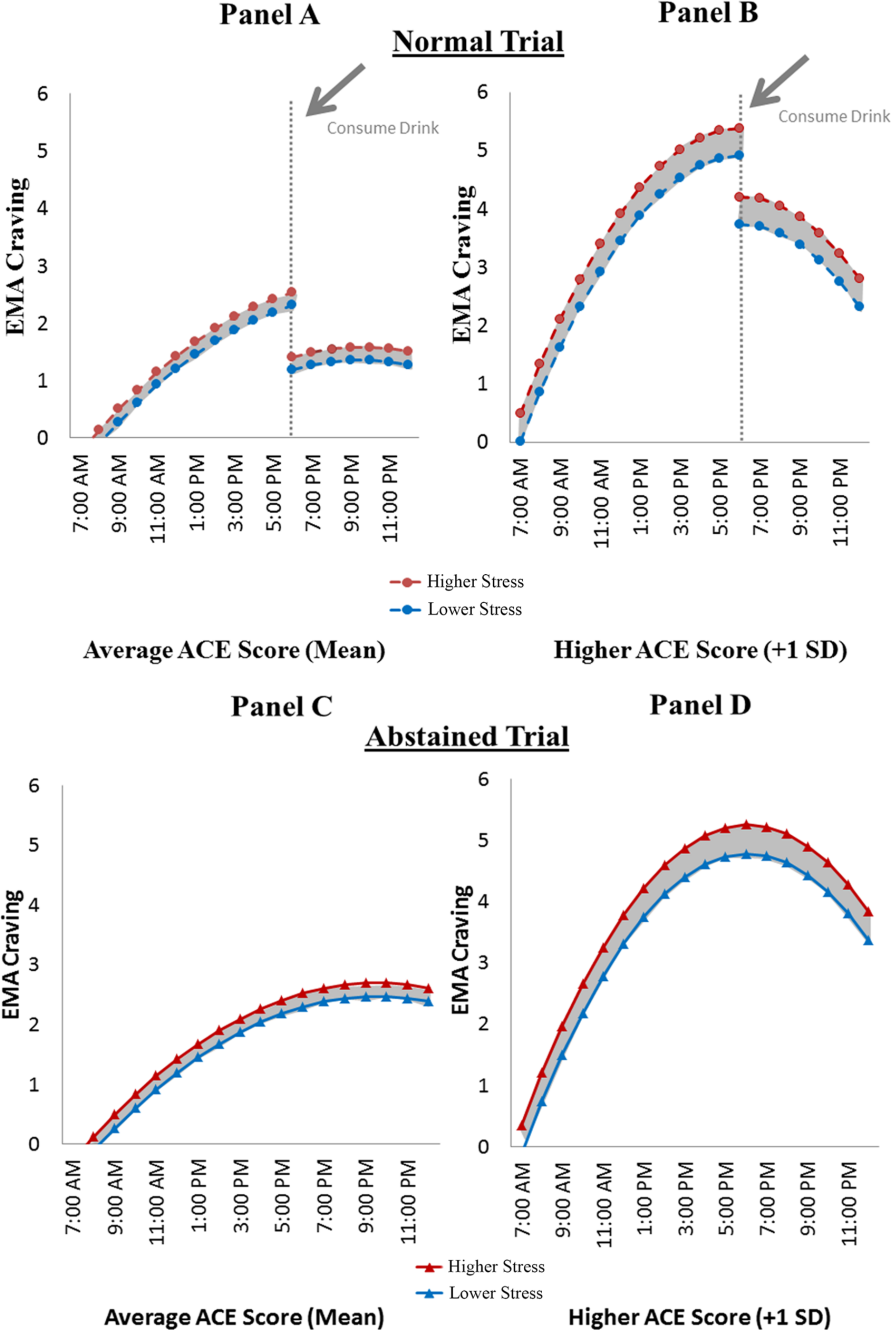
Panel A & B: Normal trial. EMA craving in higher and lower stress in average and higher ACE scores. Predicted craving scores during the normal trial as a function of time of day, higher and lower EMA stress, and alcohol consumption for individuals reporting average (Panels A) and higher (Panel B) ACE scores. Higher ACE scores exaggerated the relationship between stress and craving. Panel C & D: Abstained trial. EMA craving in higher and lower stress in average and higher ACE scores. Predicted craving scores during the abstained trial as a function of time of day and higher and lower EMA craving for individuals reporting average (Panel C) and higher (Panel D) ACE scores. Higher ACE scores exaggerated the relationship between stress and craving.

The final set of analyses that combined time of day, alcohol consumption, and stress as predictors of craving while accounting the moderating influence of ACE scores replicated and extended the findings observed in prior analyses. That is, individuals with higher ACE scores reported higher craving throughout the day (b = 1.07, SE = 0.17, p<0.001) and reached higher levels at midday (moderating effect of linear trend: b = 0.03, SE = 0.01, p<0.02); moderating effect of the quadratic trend: -0.01, SE = 0.002, p<0.001), and all participants regardless of ACE scores reported similar decreases in craving (b = -1.11, SE = 0.18, p<0.001) as a result of drinking (b = -0.03, SE = 0.15, p = 0.84). However, although higher stress was associated with greater craving (b = 0.22, SE = 0.03, p<0.001), this association was stronger among those with higher ACE scores (b = 0.11, SE = 0.02, p<0.001; see Fig 4, Panels A and B).

## Discussion

The current study showed that, after accounting for time of day and the impact of alcohol consumption on cravings, moderate-heavy drinkers’ responses were similar during both the normal and abstained trials. During the normal trial, craving intensity increased throughout the day (pre-drinking) followed by alcohol consumption that significantly lowered craving intensity (Fig 2). These results are consistent with the EI Theory of Desire which posits that substance related cues have an additive effect on craving [9]. In other words, as moderate-heavy drinkers move throughout the day, they are likely exposed to a greater number of cues that increase the probability of triggering intrusive, craving related thoughts that lead to rumination about the target of desire (alcohol). The imposed period of abstinence did not enhance craving, a finding that was contrary to our original expectation. However, according to the EI Theory of Desire, when individuals expect consumption to be delayed, they engage in strategies to reduce the discomfort associated with craving [9]. Hence, one interpretation of our finding is that, because participants knew that the abstinence period was only 3-days, they suppressed cravings during this period.

It is important to emphasize that individuals scoring higher on the ACE became more distinct from the rest of the group when considering EMA stress responses (Fig 3). As expected, as a group, moderate-heavy drinkers exhibited an inverted-U pattern of stress throughout the day. However, individuals with higher ACE scores reported significantly higher levels of stress and a steeper increase in stress at midday compared to ratings for the overall group. Interestingly, compared to abstained days, those with higher ACE scores reported higher stress on days they anticipated having a drink, but did not enjoy the same relief from craving post-drink as those with average ACE scores. The fact that individuals with higher ACE scores exhibited an elevated pre-drinking stress response on the normal as compared to the abstinence trial is interesting considering that the anticipation of consumption alone can trigger craving [9, 21]. In addition, the EI Theory of Desire posits that craving can enhance negative emotions that accompany stress due to increased attention on the state of deprivation, which may explain the elevated pre-drinking stress levels observed during abstinence in those with higher ACE scores. Also of note, Fig 3 illustrates that *post*-drinking stress levels in those with higher ACE scores remained similar to levels during abstained trials in the overall group.

Most importantly, higher ACE scores were associated with a significantly stronger stress-craving relationship in EMA response patterns (Fig 4). The EI Theory of Desire describes negative affect as a crucial component of the experience of craving, with stronger craving elicited in the context of negative emotion and cognitive elaboration more likely when the intrusive, craving related thoughts have stronger ties to negative affect [9]. Negative affect can increase the motivation for behaviors aimed at returning to a positive hedonic state and thus increase drinking behavior [22–24]. Stress often precipitates and can intensify negative affect and has been found to increase craving in alcohol dependent individuals, but not in light-moderate drinkers [25]. This suggests that craving in the presence of stress may be an important indicator of AUD risk, particularly for individuals with higher ACE scores. Greater stress related increases in craving were also found to be associated with decreased treatment outcomes in recovering alcohol dependent individuals [26], further illustrating the importance of understanding the stress-craving relationship. Although previous work has also shown conflicting results for the role of stress in alcohol craving and consumption [12], the current findings suggest that momentary stress may be more closely linked to craving in moderate-heavy drinkers who regularly experience frequent craving (higher ACE scores), providing insight to one potential path to AUD.

Strengths of this study including contrasting stress and craving during normal and abstained trials using random EMA surveys taken across the day, as well as directly before the 1^st^ drink and after the final drink of the day during the normal drinking trial. However, there are some limitations to our study design. As cue exposure has been associated with craving [12, 27, 28], assessing these variables during the time of craving ratings may have provided additional insight into our findings. Additionally, the random alerts only occurred between the hours of 9am–9pm to avoid disruption of participants’ sleep. On some occasions, this may have led to a lapse in data sampling depending on the participant’s sleep schedule. However, the participant triggered alerts (i.e. “I am going to sleep”) served to provide additional data outside the random alert schedule. In addition, this study’s somewhat selective recruitment criteria are a consideration when generalizing these findings to the population. To our knowledge, this study design (imposed period of alcohol abstinence in contrast to normal drinking routine) is new to the alcohol literature. The three consecutive days of imposed abstinence was as a starting point, but craving across the day may have increased with additional consecutive days of alcohol abstinence. It would be interesting for future work to build on these findings by extending the abstinence period. A longitudinal study including reassessment of craving/stress and drinking behavior over time would help confirm our findings and the implications they may have for risk of AUD. It is also important to note that the use of the labels “high” or “higher” ACE scores in this sample refers to scores in a sample of this demographic and should not be taken to represent high scores on the ACE when considering the entire range of possible scores. Finally, future research should assess negative affect in conjunction with craving responses to better understand how negative affect tracks with increases in craving, albeit Kavanagh and colleagues [9] view craving as a negative emotion.

In summary, EMA stress and craving throughout the day varied within this sample of moderate-heavy drinkers. Those with higher ACE scores reported a stepper increase in craving and stress throughout the day, elevated stress pre-drinking, a return to abstained levels of stress post-drinking, and a stronger stress-craving relationship compared to the rest of the group. These findings suggest that the frequency with which moderate-heavy drinkers experience craving is important to understanding patterns of stress and craving experienced across the day and may provide new insights to the vulnerability of transitioning to AUD. They also provide a baseline for which to compare how other alcohol consumption patterns may very in their experience of stress and craving.

## References

[1] Babor TF, Higgins-Biddle JC. Alcohol screening and brief intervention: dissemination strategies for medical practice and public health. Addiction. 2000;95(5):677–86. . 10885042 10.1046/j.1360-0443.2000.9556773.x

[2] Sinha R. Chronic stress, drug use, and vulnerability to addiction. Ann N Y Acad Sci. 2008;1141:105–30. doi: 10.1196/annals.1441.030 ; PubMed Central PMCID: PMCPMC2732004. 18991954 10.1196/annals.1441.030 PMC2732004

[3] Becker HC. Influence of stress associated with chronic alcohol exposure on drinking. Neuropharmacology. 2017;122:115–26. doi: 10.1016/j.neuropharm.2017.04.028 ; PubMed Central PMCID: PMCPMC5497303. 28431971 10.1016/j.neuropharm.2017.04.028 PMC5497303

[4] Breese GR, Chu K, Dayas CV, Funk D, Knapp DJ, Koob GF, et al Stress enhancement of craving during sobriety: a risk for relapse. Alcoholism, clinical and experimental research. 2005;29(2):185–95. ; PubMed Central PMCID: PMC2868509. 15714042 10.1097/01.alc.0000153544.83656.3c PMC2868509

[5] de Bruijn C, Korzec A, Koerselman F, van Den Brink W. Craving and withdrawal as core symptoms of alcohol dependence. J Nerv Ment Dis. 2004;192(7):494–502. . 15232320 10.1097/01.nmd.0000131912.71344.e4

[6] NIAAA(a). National Institute on Alcohol Abuse and Alcoholism—Alcohol Use Disorder. Available from: https://www.niaaa.nih.gov/alcohol-health/overview-alcohol-consumption/alcohol-use-disorders.

[7] Fazzino TL, Harder VS, Rose GL, Helzer JE. A daily process examination of the bidirectional relationship between craving and alcohol consumption as measured via interactive voice response. Alcoholism, clinical and experimental research. 2013;37(12):2161–7. doi: 10.1111/acer.12191 ; PubMed Central PMCID: PMCPMC3815497. 23889127 10.1111/acer.12191 PMC3815497

[8] Ray LA, Miranda R Jr., Tidey JW, McGeary JE, MacKillop J, Gwaltney CJ, et al Polymorphisms of the mu-opioid receptor and dopamine D4 receptor genes and subjective responses to alcohol in the natural environment. Journal of abnormal psychology. 2010;119(1):115–25. doi: 10.1037/a0017550 ; PubMed Central PMCID: PMCPMC3703617. 20141248 10.1037/a0017550 PMC3703617

[9] Kavanagh DJ, Andrade J, May J. Imaginary relish and exquisite torture: the elaborated intrusion theory of desire. Psychol Rev. 2005;112(2):446–67. doi: 10.1037/0033-295X.112.2.446 . 15783293 10.1037/0033-295X.112.2.446

[10] Dua JK. The role of negative affect and positive affect in stress, depression, self-esteem, assertiveness, Type A behaviors, psychological health, and physical health. Genet Soc Gen Psychol Monogr. 1993;119(4):515–52. . 8150272

[11] Cooney NL, Litt MD, Morse PA, Bauer LO, Gaupp L. Alcohol cue reactivity, negative-mood reactivity, and relapse in treated alcoholic men. J Abnorm Psychol. 1997;106(2):243–50. . 9131844 10.1037//0021-843x.106.2.243

[12] Wray TB, Merrill JE, Monti PM. Using Ecological Momentary Assessment (EMA) to Assess Situation-Level Predictors of Alcohol Use and Alcohol-Related Consequences. Alcohol Res. 2014;36(1):19–27. ; PubMed Central PMCID: PMCPMC4432855. 26258997 10.35946/arcr.v36.1.03 PMC4432855

[13] Shiffman S, Stone AA, Hufford MR. Ecological momentary assessment. Annu Rev Clin Psychol. 2008;4:1–32. . 18509902 10.1146/annurev.clinpsy.3.022806.091415

[14] Swendsen J. Contributions of mobile technologies to addiction research. Dialogues Clin Neurosci. 2016;18(2):213–21. ; PubMed Central PMCID: PMCPMC4969708. 27489461 10.31887/DCNS.2016.18.2/jswendsen PMC4969708

[15] Statham DJ, Connor JP, Kavanagh DJ, Feeney GF, Young RM, May J, et al Measuring alcohol craving: development of the Alcohol Craving Experience questionnaire. Addiction. 2011;106(7):1230–8. doi: 10.1111/j.1360-0443.2011.03442.x . 21438940 10.1111/j.1360-0443.2011.03442.x

[16] NIAAA(b). Drinking Levels Defined. Available from: https://www.niaaa.nih.gov/alcohol-health/overview-alcohol-consumption/moderate-binge-drinking.

[17] Wang MQ, Nicholson ME, Jones CS, Fitzhugh EC, Westerfield CR. Acute alcohol intoxication, body composition, and pharmacokinetics. Pharmacol Biochem Behav. 1992;43(2):641–3. . 1438504 10.1016/0091-3057(92)90205-t

[18] Sullivan JT, Sykora K, Schneiderman J, Naranjo CA, Sellers EM. Assessment of alcohol withdrawal: the revised clinical institute withdrawal assessment for alcohol scale (CIWA-Ar). Br J Addict. 1989;84(11):1353–7. . 2597811 10.1111/j.1360-0443.1989.tb00737.x

[19] Vakili S, Sobell LC, Sobell MB, Simco ER, Agrawal S. Using the Timeline Followback to determine time windows representative of annual alcohol consumption with problem drinkers. Addictive behaviors. 2008;33(9):1123–30. doi: 10.1016/j.addbeh.2008.03.009. WOS:000258046600004. 18562125 10.1016/j.addbeh.2008.03.009

[20] May J, Andrade J, Kavanagh DJ, Feeney GF, Gullo MJ, Statham DJ, et al The craving experience questionnaire: a brief, theory-based measure of consummatory desire and craving. Addiction. 2014;109(5):728–35. doi: 10.1111/add.12472 . 24400950 10.1111/add.12472

[21] Juliano LM, Brandon TH. Reactivity to instructed smoking availability and environmental cues: evidence with urge and reaction time. Exp Clin Psychopharmacol. 1998;6(1):45–53. . 9526145 10.1037//1064-1297.6.1.45

[22] Dvorak RD, Pearson MR, Sargent EM, Stevenson BL, Mfon AM. Daily associations between emotional functioning and alcohol involvement: Moderating effects of response inhibition and gender. Drug Alcohol Depen. 2016;163:S46–S53. doi: 10.1016/j.drugalcdep.2015.09.034. WOS:000378457100008. 27306731 10.1016/j.drugalcdep.2015.09.034 PMC5238712

[23] Simons JS, Dvorak RD, Batien BD, Wray TB. Event-level associations between affect, alcohol intoxication, and acute dependence symptoms: Effects of urgency, self-control, and drinking experience. Addict Behav. 2010;35(12):1045–53. doi: 10.1016/j.addbeh.2010.07.001. WOS:000283404500001. 20685044 10.1016/j.addbeh.2010.07.001 PMC3298685

[24] Epler AJ, Tomko RL, Piasecki TM, Wood PK, Sher KJ, Shiffman S, et al Does Hangover Influence the Time to Next Drink? An Investigation Using EcologicalMomentary Assessment. Alcoholism-Clinical and Experimental Research. 2014;38(5):1461–9. doi: 10.1111/acer.12386. WOS:000334657200034. 24588377 10.1111/acer.12386 PMC3999207

[25] Sinha R, Fox HC, Hong KA, Bergquist K, Bhagwagar Z, Siedlarz KM. Enhanced negative emotion and alcohol craving, and altered physiological responses following stress and cue exposure in alcohol dependent individuals. Neuropsychopharmacol. 2009;34(5):1198–208. doi: 10.1038/npp.2008.78 ; PubMed Central PMCID: PMCPMC2734452. 18563062 10.1038/npp.2008.78 PMC2734452

[26] Higley AE, Crane NA, Spadoni AD, Quello SB, Goodell V, Mason BJ. Craving in response to stress induction in a human laboratory paradigm predicts treatment outcome in alcohol-dependent individuals. Psychopharmacology (Berl). 2011;218(1):121–9. doi: 10.1007/s00213-011-2355-8 ; PubMed Central PMCID: PMC3191263. 21607563 10.1007/s00213-011-2355-8 PMC3191263

[27] Serre F, Fatseas M, Swendsen J, Auriacombe M. Ecological momentary assessment in the investigation of craving and substance use in daily life: a systematic review. Drug Alcohol Depend. 2015;148:1–20. doi: 10.1016/j.drugalcdep.2014.12.024 . 25637078 10.1016/j.drugalcdep.2014.12.024

[28] Miranda R, Ray L, Blanchard A, Reynolds EK, Monti PM, Chun T, et al Effects of naltrexone on adolescent alcohol cue reactivity and sensitivity: an initial randomized trial. Addict Biol. 2014;19(5):941–54. doi: 10.1111/adb.12050 ; PubMed Central PMCID: PMCPMC3729253. 23489253 10.1111/adb.12050 PMC3729253

